# A fault diagnosis method based on Auxiliary Classifier Generative Adversarial Network for rolling bearing

**DOI:** 10.1371/journal.pone.0246905

**Published:** 2021-03-01

**Authors:** Chunming Wu, Zhou Zeng

**Affiliations:** 1 Key Laboratory of Modern Power System Simulation and Control & Renewable Energy Technology, Ministry of Education, Northeast Electric Power University, Jilin, China; 2 Department of Electrical Engineering, Northeast Electric Power University, Jilin, China; Polytechnical Universidad de Madrid, SPAIN

## Abstract

Rolling bearing fault diagnosis is one of the challenging tasks and hot research topics in the condition monitoring and fault diagnosis of rotating machinery. However, in practical engineering applications, the working conditions of rotating machinery are various, and it is difficult to extract the effective features of early fault due to the vibration signal accompanied by high background noise pollution, and there are only a small number of fault samples for fault diagnosis, which leads to the significant decline of diagnostic performance. In order to solve above problems, by combining Auxiliary Classifier Generative Adversarial Network (ACGAN) and Stacked Denoising Auto Encoder (SDAE), a novel method is proposed for fault diagnosis. Among them, during the process of training the ACGAN-SDAE, the generator and discriminator are alternately optimized through the adversarial learning mechanism, which makes the model have significant diagnostic accuracy and generalization ability. The experimental results show that our proposed ACGAN-SDAE can maintain a high diagnosis accuracy under small fault samples, and have the best adaptation performance across different load domains and better anti-noise performance.

## 1 Introduction

As a common part of rotating machinery, rolling bearing may cause great economic loss if it breaks down in the working process [1]. Therefore, it is of great significance for the normal operation of the machine to diagnose the rolling bearing effectively [2]. At present, vibration signal analysis is one of the widely employed and effective techniques for machinery fault diagnosis and health monitoring [3]. In essence, machinery fault diagnosis can be regarded as a pattern recognition problem, which includes data acquisition, feature extraction and fault classification. The diagnostic performance largely depends on the effectiveness of feature extraction and classification methods. Thus, traditional fault diagnosis methods based on vibration are generally: signal processing methods based on time domain, frequency domain and time-frequency domain. These methods include time domain statistics, short-time Fourier transform [4], wavelet transform [5], Empirical Mode Decomposition (EMD) [6], Hilbert-Huang Transform (HHT) [7] and other variants [8–11]. Then, these extracted features are fed into some shallow machine learning algorithms, including Artificial Neural Network (ANN) [12], Support Vector Machine (SVM) [13], cluster analysis [14], etc. However, the fault feature representation extracted from the above methods is usually designed manually and requires a lot of professional knowledge and manpower [15]. At the same time, most of the methods are limited to the domain and can not be well extended to other new fault diagnosis fields. Instead, deep learning can effectively solve the above problems by modeling the high-level representation of data and predicting/classifying patterns through a layered architecture of multiple nonlinear processing units [16].

Since Hinton et al. [17] proposed unsupervised layer by layer training combined with supervised fine-tuning method, deep learning theory has become a hot spot in the field of machine learning and artificial intelligence, and has made brilliant achievements in computer vision, speech recognition and other fields. Some experts and scholars have also applied for deep learning theory to the field of mechanical fault diagnosis. Chen et al. [18] proposed a bearing fault diagnosis method based on Deep Belief Network (DBN). By using the advantages of DBN automatic feature extraction and classification, the original vibration signal is directly studied and stratified training, and the fault diagnosis results are automatically given. Considering the multi-scale characteristics inherent in vibration signals of a gearbox, Jiang et al. [19] proposed a new multi-scale convolutional neural network (MSCNN) architecture that can perform multi-scale features extraction and classification simultaneously. Due to the time-series characteristics of wind turbine vibration signals, Lei et al. [20] adopted the Long Short-Term Memory (LSTM) model to realize the end-to-end fault diagnosis of wind turbines.

Although the above fault methods can achieve good results in some specific aspects, there is still room for improvement: (i) Most of the improvements in the traditional depth model to make the model satisfy better diagnostic accuracy for specific data sets, but it may not be suitable for practical fault diagnosis tasks. (ii) In fact, the machinery generally runs under normal working conditions for a long time, and the sensor can collect enough positive samples, while the negative samples collected under fault conditions are seriously unbalanced compared with the positive samples. Therefore, the diagnosis performance of the unbalanced data set under small sample conditions is very poor. (iii) At the same time, considering the cross domain adaptive problem with the actual variable load conditions and the influence of high background noise pollution, the diagnostic performance of the model will be further deteriorated.

Goodfellow et al. [21] proposed a Generative Adversarial Network(GAN) in 2014. Due to its powerful performance, GAN has made great achievements in the field of image processing. Radford et al. [22] proposed a novel Deep Convolution Generative Adversarial Network (DCGAN) based on the GAN, which is stable in the training process and can generate high-quality images.The application of GAN to the field of mechanical fault diagnosis provides a new perspective. Shao et al. [23] applied GAN as a data set enhancement technique to fault diagnosis for small fault sample sizes, and achieved good results. Han et al. [24] proposed a novel deep adversarial convolutional neural network (DACNN) framework. By introducing adversarial learning into CNN, it helps to make feature representation more robuster and enhance the generalization ability of the training model. Zhao et al. [25] proposed an improved Wassertein GAN fault diagnosis method based on K-means and applied it to aero-engine fault diagnosis. Wassertein GAN with gradient penalty was used to make the model converge process faster.

Aiming at the problems that the data unbalances caused by small fault sample size, cross domain adaptive problem under variable load and the influence of high background noise pollution in the fault diagnosis of rolling bearing, we proposed a novel fault diagnosis method of rolling bearing which combines ACGAN and SDAE. Different from the traditional GAN, this paper introduces the variant structure ACGAN with auxiliary classification labels. In detail, we use one-dimensional convolutional neural network (1D-CNN) as a generator, and use category labels as auxiliary information to enhance the original GAN, improve the generation effect of the generator, and generate high-quality labeled artificial samples to expand the number of fault samples. The SDAE is used as a discriminator to identify the authenticity and fault of the input samples. SDAE can automatically extract features with better robustness by adding noise to samples for sample reconstruction. At the same time, in the process of simulating the generation of false data, the generator is helpful to understand the distribution of original data, and the adversarial learning is used as a cross domain regularizer to learn the universal and domain-invariant features of data.

The rest of this paper is organized as follows: in section 2, we will introduce the theoretical background of the relevant methods. Section 3 details our proposed ACGAN-SDAE method. In section 4, some experiments are performed to evaluate our method and other methods. Finally, section 5 summarizes the full text.

## 2 The theoretical background of related methods

### 2.1 SDAE

**Fig 1:**
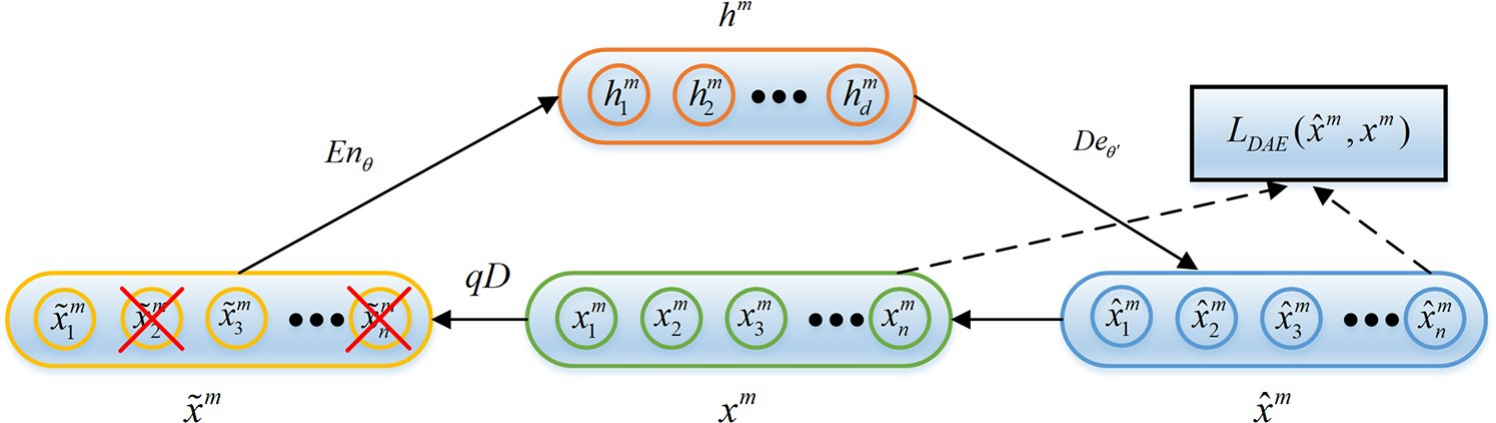
The structure principle of DAE.

SDAE [26] consists of multiple Denoising Auto Encoder (DAE) [27] by stacking. Like the standard Auto Encoder (AE), DAE consists of encoder and decoder. But different from standard AE, DAE enhances the robustness of extracted features by adding impairment noise to the input data, thereby enhancing its anti-noise ability. The encoder compresses the input data in the high-dimensional space to obtain the encode vectors in the low-dimensional space. The decoder reconstructs the encode vectors to obtain the original input data without noise. The structure principle of standard DAE is shown as **Fig 1**.

Given an unlabeled rolling bearing faults sample training set ${\left\{x^{m}\right\}}_{m=1}^{M}$, the noise *qD* is added to the training sample *x*^*m*^ before coding to get the sample with noise ${\tilde{x}}^{m}$.

$${\tilde{x}}^{m}∼qD\left({\tilde{x}}^{m}|x^{m}\right)$$ (1)

where *qD* is a binomial random hidden noise.

The coding network encodes the sample with noise ${\tilde{x}}^{m}$. In the coding process, the encode function *En*_*θ*_ maps the samples ${\tilde{x}}^{m}$ to the encode vectors *h*^*m*^.

$$h^{m}=En_{\theta }({\tilde{x}}^{m})=\sigma (w_{e}{\tilde{x}}^{m}+b_{e})$$ (2)

where *σ* is the sigmoid activation function and *σ* = 1/(1+exp(−*x*)), *θ* is the parameters set of the encoding network and *θ* = {*w*_*e*_,*b*_*e*_}, *w*_*e*_ and *b*_*e*_ are the weight matrix and bias vector of the coding network respectively.

The decoding network reversely transforms the encode vectors *h*^*m*^ into the reconstructed representation *x*^*m*^ of ${\hat{x}}^{m}$ by decode function *De*_*θ*′_.

$${\hat{x}}^{m}=De_{\theta^{\prime }}(h^{m})=\sigma (w_{d}h^{m}+b_{d})$$ (3)

where *θ*′ is the parameters set of the decoding network and *θ*′ = {*w*_*d*_,*b*_*d*_}, *w*_*d*_ and *b*_*d*_ are the weight matrix and bias vector of the decoding network respectively.

DAE aims to complete the training of the whole network by optimizing the parameters set Θ = {*θ*,*θ*′} to minimize the reconstruction error $L_{DAE}({\hat{x}}^{m},x^{m})$ between ${\hat{x}}^{m}$ and *x*^*m*^.

$$L_{DAE}({\hat{x}}^{m},x^{m})=\underset{\Theta }{\arg}\;\min\frac{1}{M}{\sum_{m=1}^{M}\left\|{\hat{x}}^{m}-x^{m}\right\|}^{2}$$ (4)

where Θ is a set of parameters for DAE and Θ = {*θ*,*θ*′}, *M* is the sample size.

**Fig 2:**
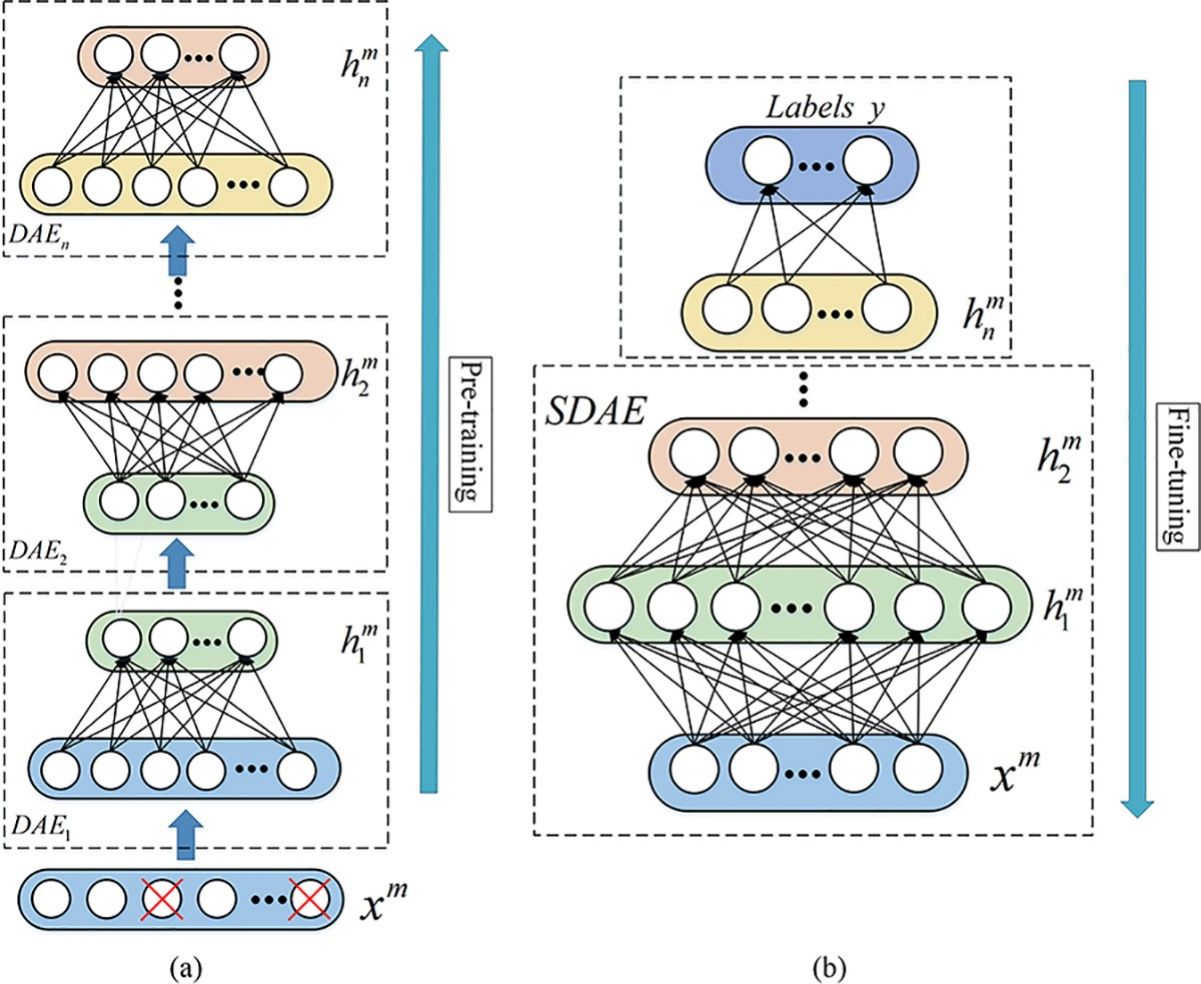
The structure principle of SDAE. (a) Pre-training, (b) Fine-tuning.

SDAE constructs deep networks by stacking multiple DAEs, and extracts deep features through unsupervised learning. SDAE training steps includes pre-training and fine-tuning, as shown in **Fig 2**. The pre-training step trains each layer of DAE through unsupervised layer-by-layer greedy learning to extract sample fault features. The encode vector of the hidden layer of the previous layer of DAE is used as the input to next layer of DAE, and repeat this process until the last layer of DAE_n_ is trained and the encoding vector $h_{n}^{m}$ is obtained. Finally, supervised fine-tuning is carried out by using labeled sample data and adding a Solfmax classifier at the top of the network.

### 2.2 GAN

**Fig 3:**
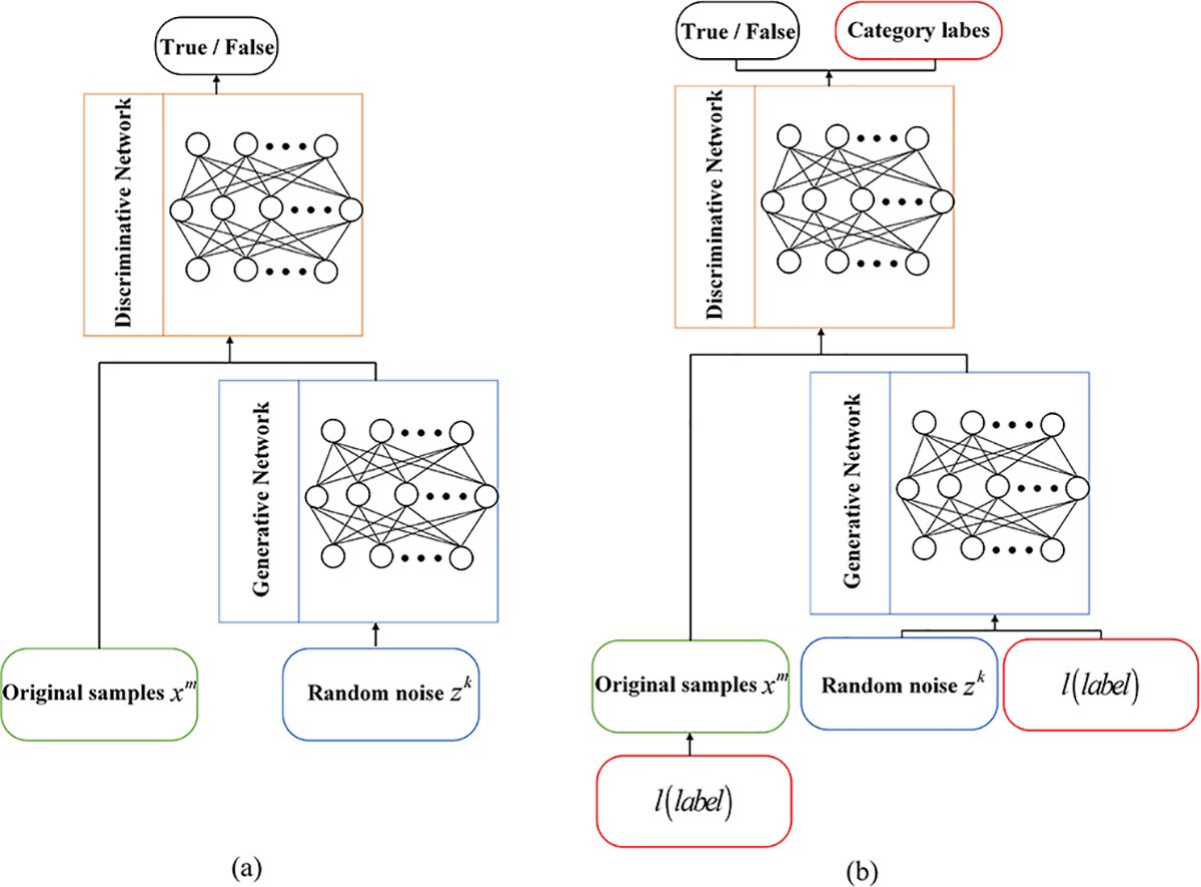
The structure of (a) reguar GAN, and (b) ACGAN.

The structure of GAN is inspired by game theory, regular GAN consists of two parts: generator *G* and discriminator *D* (as shown in **Fig 3A**). Among them, *x*^*m*^ is sampled from the original sample and *z*^*k*^ is the input of the generator. The generator aims to capture the potential distribution of real data samples *x*^*m*^ and generate realistic generated data *G*(*z*^*k*^) from Gaussian random noise vector *z*^*k*^ in an attempt to deceive the discriminator. Instead, the purpose of the discriminator is to distinguish whether the input data is real data *x*^*m*^ or generated data *G*(*z*^*k*^).

GAN continuosly optimizes the generation ability of *G* and the discrimination ability of *D* through the adversarial learning mechanism, and finally they reach the Nash equilibrium. The optimization process is a minimax two-player game that can be formulated as:

$$\begin{array}{l} \underset{G}{\min}\;\underset{D}{\max}V\left(D,G\right)=E_{x\sim p_{data}\left(x\right)}\left[\log D\left(x^{m}\right)\right]\\ \;+E_{z\sim p_{z}\left(z\right)}\left[\log\left(1-D\left(G\left(z^{k}\right)\right)\right)\right] \end{array}$$ (5)

where *p*_*data*_(*x*) is the real data distribution, *p*_*z*_(z) is the prior distribution of the noise vector *z*, $E_{x\sim p_{data}(x)}$ is the expected value of the real data distribution of *x*, and $E_{z\sim p_{z}(z)}$ is the expected value of *z* sampled from the noise.

In the process of training, one part is fixed and the parameters of the other network are updated. Training *D* maximizes log*D*(*x*^*m*^) and training *G* minimizes log(1−*D*(*G*(*z*^*k*^))). The generator defines a probability distribution function *p*_*g*_, and GAN expects *p*_*g*_ to converge to the real data distribution *p*_*data*_ through alternating iteration. If and only if *p*_*g*_ = *p*_*data*_ reaches Nash equilibrium, GAN can well estimate the actual distribution of real samples and generate new samples to expand the training fault sample set.

### 2.3 ACGAN

Odena et al. [28] proposed a variant architecture of regular GAN to achieve accurate classification of images in the MNIST dataset. This variant architecture is called Auxiliary Classifier Generative Adversarial Network (ACGAN) by adding category labels to generator and discriminator(as shown in **Fig 3B**). According to research, when GAN is allowed to process additional information, the original generation task of the model will be completed better. Therefore, high quality generated samples can be generated by using auxiliary category label information.

For the generator, there are two inputs: random noise vector *z* and label classification information *c*. And the generated data is *X*_*fake*_ = *G*(*c*,*z*). For the discriminator, it is necessary to judge whether the data source is the probability distribution of the real data and the probability distribution of the data source for the classification label, so that the discriminator can not only identify the data source but also distinguish various fault categories. Therefore, the objective function of ACGAN consists of two parts, as shown in the following formula:

$$\begin{array}{l} L_{s}=E_{x\sim P_{data}}\left[\log P\left(source=real|x_{real}\right)\right]\\ \;+E_{z\sim P_{z}}\left[\log P\left(source=fake|x_{fake}\right)\right] \end{array}$$ (6)

$$\begin{array}{l} L_{c}=E_{x\sim P_{data}}\left[\log P\left(class=c|x_{real}\right)\right]\\ \;+E_{z\sim P_{z}}\left[\log P\left(class=c|x_{fake}\right)\right] \end{array}$$ (7)

The first part *L*_*s*_ is a cost function for the truthfulness of the data, and the second part *L*_*c*_ is a cost function for the accuracy of data classification. In the training process, the optimization direction is to train the discriminator to maximize *L*_*s*_+*L*_*c*_, and the generator to minimize *L*_*s*_−*L*_*c*_. The corresponding physical meaning is that the discriminator is called upon to distinguish between real data and generated data as far as possible and to classify the data effectively. For the generator, the generated data are considered as real data as possible and the data can be classified effectively.

## 3. The proposed fault diagnosis method

In this paper, aiming at the data unbalance problem caused by the small fault sample size in the actual rolling bearing fault diagnosis, the cross domain adaptive problem with variable load conditions and the influence of high background noise pollution, a novel ACGAN-SDAE fault diagnosis method is proposed.

### 3.1 Fault diagnosis model of ACGAN-SDAE

**Fig 4:**
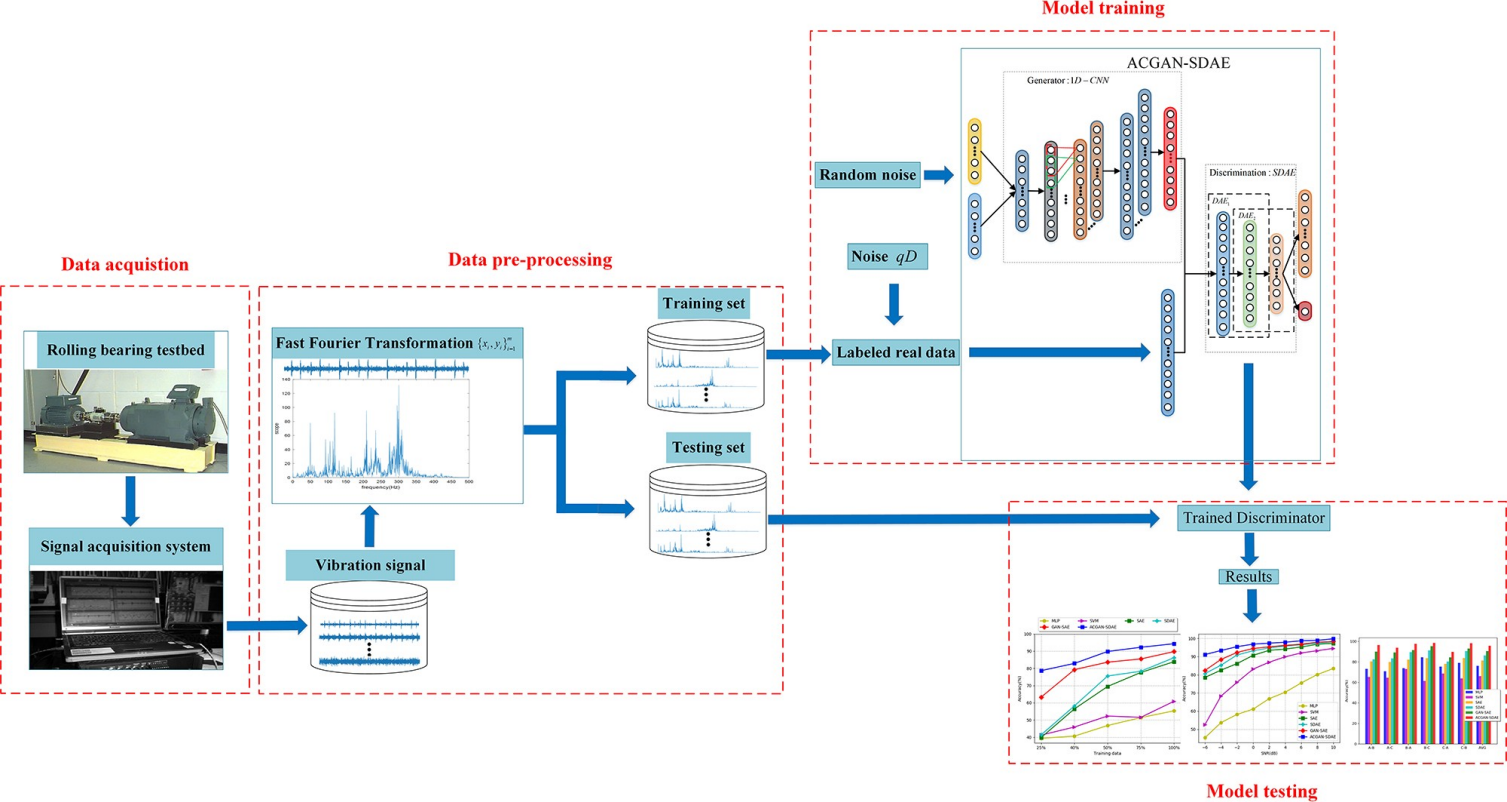
The overall architecture of ACGAN-SDAE fault diagnosis model.

In this paper, by combining ACGAN and SDAE, we proposed an ACGAN-SDAE fault diagnosis method. The overall structure of the model as shown in **Fig 4**. In detail, an one-dimensiona convolutional neural network (1D-CNN) [29] is used as the generator, and use category labels as auxiliary information to enhance the original GAN, improve the generation effect of the generator, and generate high-quality labeled artificial samples to expand the number of fault samples. By adding category labels, the generator can generate specific conditional data and make model training more stable. The SDAE is used as a discriminator to distinguish the authenticity and fault category of the input samples.

### 3.2 Training of discriminator

**Fig 5:**
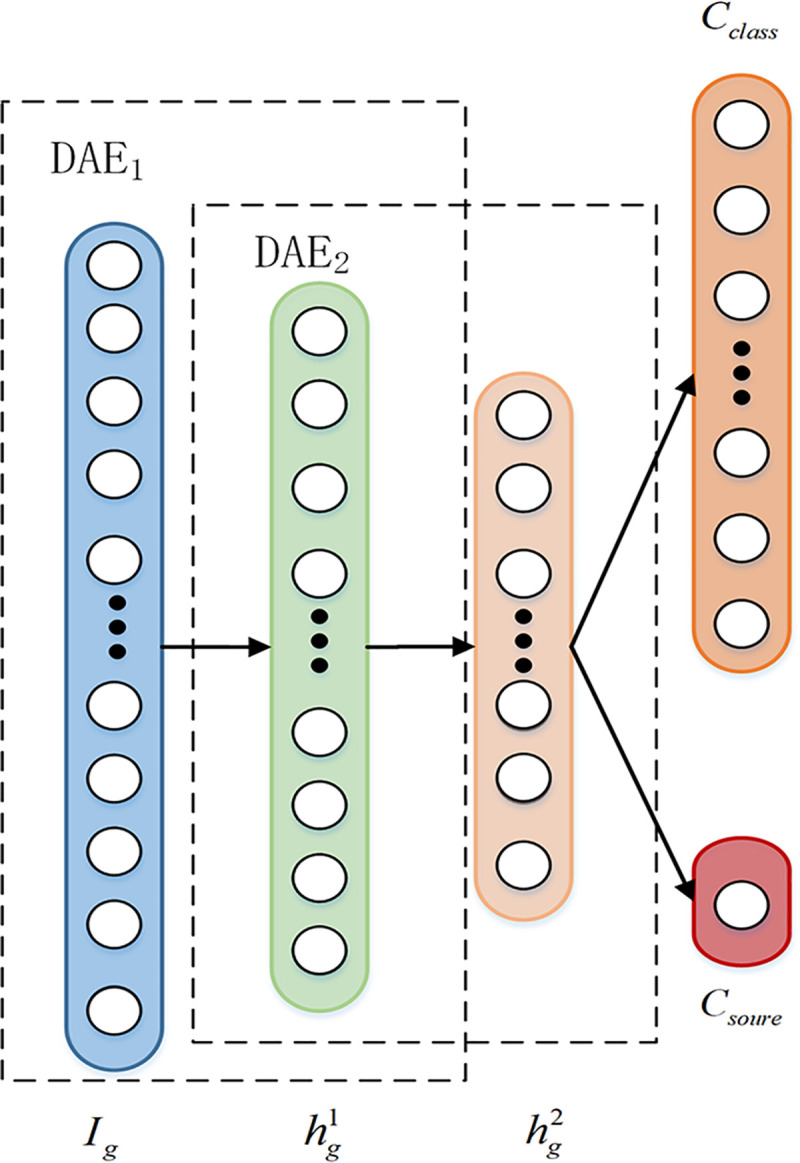
Network structure of discriminator.

The four-layer structure of the generator SDAE is 1024-800-200-10 (1), as shown in **Fig 5**. The generated samples ${\left\{x_{fake}^{k}\right\}}_{k=1}^{K}$ are labeled as 0, and the corresponding real category labels are $y_{fake}^{k}$. The real samples ${\left\{x_{real}^{m}\right\}}_{m=1}^{M}$ are labeled as 1, and the corresponding real category labels are $y_{real}^{m}$. Then input them together to the discriminator SDAE for authenticity discrimination and fault identification. SDAE adds two label classifiers in the top layer. The sigmoid function is used to predict the sample source, and output the corresponding authenticity labels $d_{real}^{m}$, $d_{fake}^{k}$. The solfmax function is used to predict the fault category labels $c_{real}^{m}$, $c_{fake}^{k}$.

ACGAN-SDAE completes the training of discriminator by minimizing the error of authenticity labels and fault category labels through (10).

$$\begin{array}{l} L_{c}=-\frac{1}{M}\sum_{m=1}^{M}\left[y_{real}^{m}\ln c_{real}^{m}+\left(1-y_{real}^{m}\right)\ln\left(1-c_{real}^{m}\right)\right]\\ \;-\frac{1}{K}\sum_{k=1}^{K}\left[y_{fake}^{k}\ln c_{fake}^{k}+\left(1-y_{fake}^{k}\right)\ln\left(1-c_{fake}^{k}\right)\right] \end{array}$$ (8)

$$L_{d}=-\frac{1}{M}\sum_{m=1}^{M}\ln d_{real}^{m}-\frac{1}{K}\sum_{k=1}^{K}\ln\left(1-d_{fake}^{k}\right)$$ (9)

$$L_{D}=\underset{\Theta_{D}}{\arg\;\min}\left(L_{c}+L_{d}\right)$$ (10)

where *L*_*D*_ is the loss function of the discrimination in ACGAN-SDAE, Θ_*D*_ is the parameter set, *L*_*c*_ is the cross-entropy loss error of the category label, *L*_*d*_ is the cross-entropy loss error of the authenticity label.

### 3.3 Training of generator

**Fig 6:**
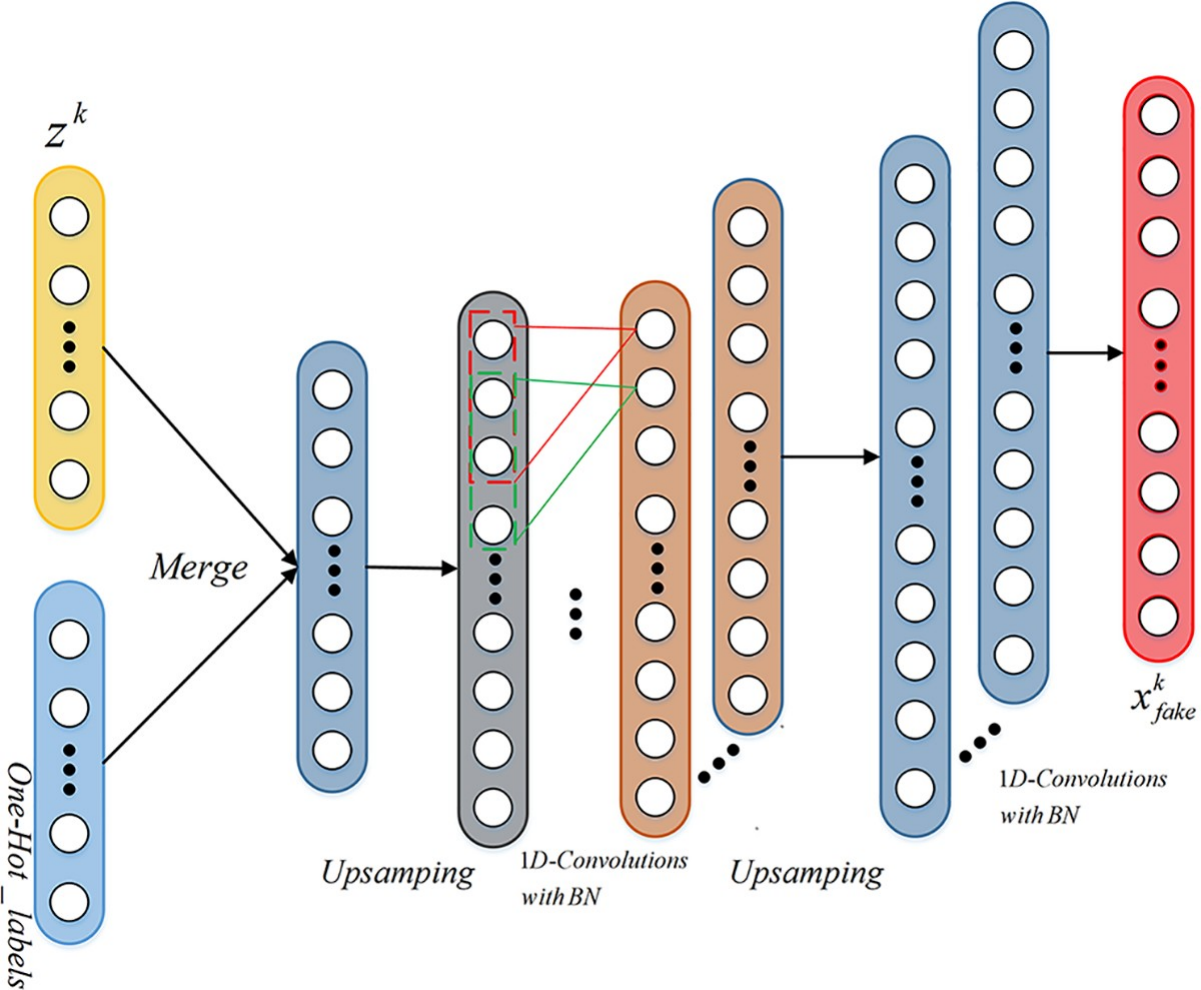
Network structure of generator.

The generator adopts 1D-convolution operation, in which the first layer is the input layer, which is a combination of Gaussian random noise vector input and category input, and contains two up-sampling layers of size 2. Then two layers of convolution are performed separately, and each layer uses batch normalization, and its momentum is 0.8. The kernel size of the first 1D convolutional layer is 16, has 16 feature maps, and uses the Rectified Linear Unit(ReLu) activation function. The second 1D fusion layer has a kernel size of 8, including 1 feature map, and uses the hyperbolic tangent function as the activation function. Its network structure is shown as in **Fig 6**. The output of the generator is one-dimensional data sample.

The generated sample ${\left\{x_{fake}^{k}\right\}}_{k=1}^{K}$ are labeled as 1 and input it to discriminator SDAE for authenticity verification. Then complete the training of the generator by minimizing the (12).

$$L_{g}=-\frac{1}{K}\sum_{k=1}^{K}\ln d_{fake}^{k}$$ (11)

$$L_{G}=\underset{\Theta_{G}}{\mathrm{argmin}}\left(L_{c}+L_{g}\right)$$ (12)

where *L*_*G*_ is the loss function of the generator in ACGAN-SDAE, Θ_*G*_ is the parameter set, *L*_*g*_ is the cross-entropy loss error of the authenticity label.

### 3.4 Adversarial training mechanism of model

The model realizes the adversarial training mechanism by alternately optimizing the generator and the discriminator. Through the zero-sum game between the them, its optimization goal has been passed as a minimum-maximization problem. Based on the above loss function, the ADAM optimizer is used for training, the learning rate of the discriminator is 0.001, the learning rate of the generator is 0.002, and updates parameters iteratively. The training process can be divided into three steps.

*Step 1*: Firstly, generator generates fake samples from Gaussian random noise of potential space with class labels.
*Step 2*: Then, the generated samples and the original samples are input into the generator SDAE for authenticity identification and fault classification. By training the above loss function, the labels and parameters in the discriminator are able to be updated.
*Step 3*: After training the discriminator, at this stage, the discriminator is set to be untrainable and its parameters are frozen. In this stage, only the parameters in generator can be updated, and generator can be trained to generate more realistic fake samples. After a period is completed, the training process starts again from *Step 1*.

Through the above multiple alternating optimization iterations, until the generator and discriminator reach Nash equilibrium, the training of the whole model is accomplished.

### 3.5 Implementation of fault diagnosis algorithm

**Fig 7:**
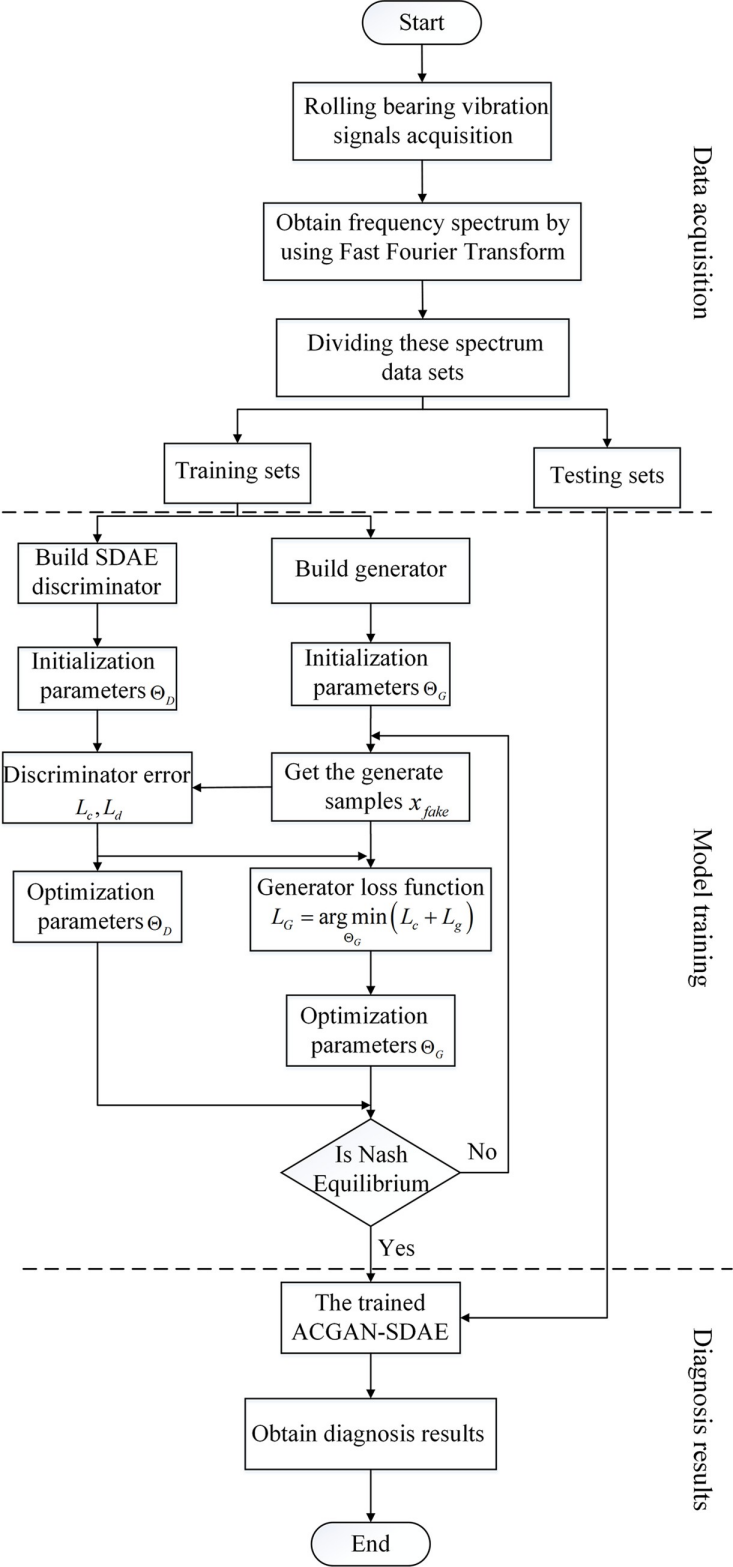
The flowchart of ACGAN-SDAE fault diagnosis method.

The steps of fault diagnosis in this paper are mainly divided into three parts: data acquisition and pre-processing, model training, fault identification. The algorithm flow chart is shown in **Fig 7**.

1. **Data acquisition and pre-processing:** In the rolling bearing feature extraction process, considering the complexity of the original vibration signal, the spectral signal is used as the input signal of the model. Firstly, a sensor is used to collect the original vibration signal of the rolling bearing, and the frequency spectrum sample ${\left\{x_{i},y_{i}\right\}}_{i=1}^{m}$ is obtained through the Fast Fourier Transform (FFT), which is divided into training set and testing set.
2. **Model training:** The training set is input into the ACGAN-SDAE model. Through the adversarial training of generator and discriminator, the training of the whole model is completed by using ADMA optimizer, alternating optimization generator and discriminator until the they reaches Nash equilibrium.
3. **Fault identification:** The testing set is input into the trained discriminator SDAE, and output the diagnosis results.

## 4. Experimental results and analysis

### 4.1 Dataset description

**Fig 8:**
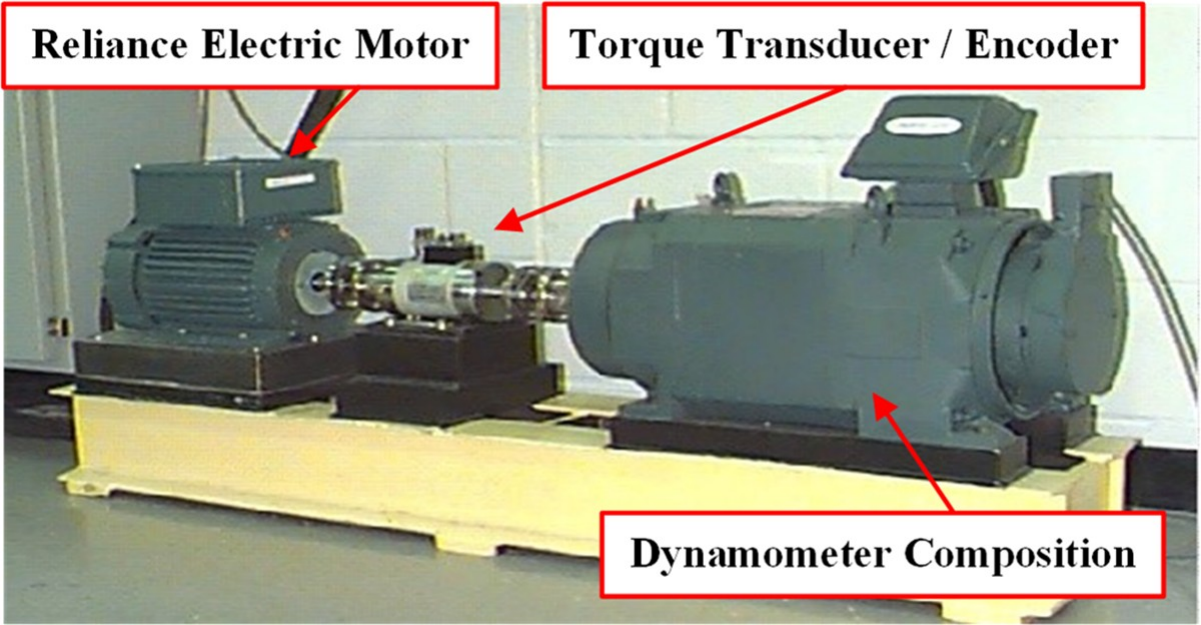
Experiment platform.

**Fig 9:**
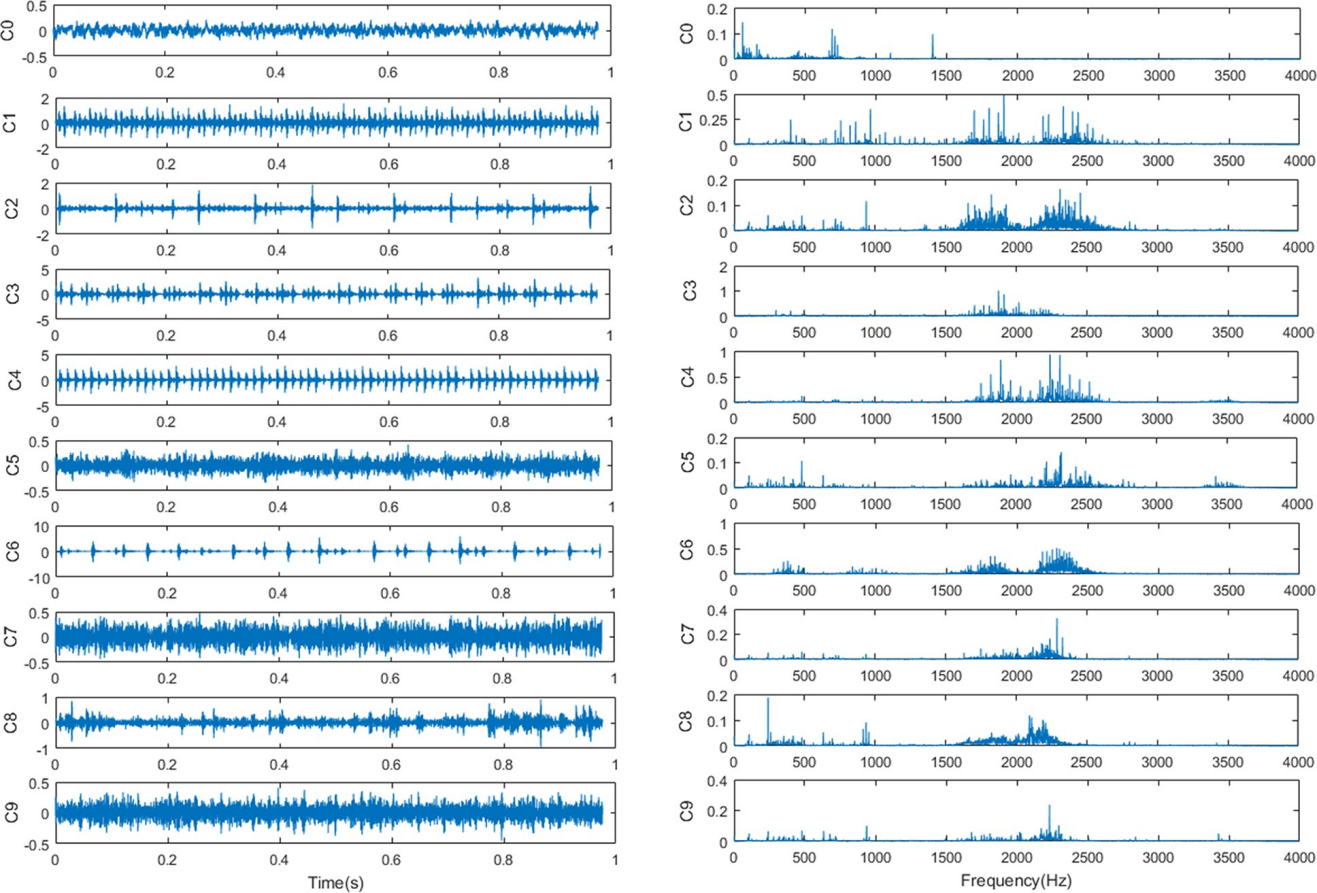
The original vibration signals in the 10 health conditions. (a) the time-domain waveform and (b) the corresponding frequency spectra.

The CWRU rolling bearing data set is obtained by the Electrical Engineering Laboratory of Case Western Reserve University [30]. It is an open data set and widely used in fault diagnosis and it can be obtained through the website: https://csegroups.case.edu/bearingdatacenter. The experimental platform is shown in **Fig 8**. The vibration data used in this study was collected from the driving end of the motor at three speeds of 1750rpm, 1772rpm and 1797rpm. And its sampling frequency was 12kHz. The bearing with fault was machined by EDM, which caused different degrees of damage to the inner race, outer race and roller of the bearing. The damage diameter included 0.007 inches, 0.014 inches and 0.021 inches, with a total of 9 damage states. Therefore, the data contains four health states: 1) Normal condition (Normal) 2) Inner race fault (IF) 3) Outer race fault (OF) 4) Roller fault (RF). Typical time-domain waveform and frequency spectra of the original vibration signals in the 10 health conditions are shown in **Fig 9**.

In the experiment, FFT is used to preprocess the original signal to obtain spectrum samples, and 1024 data points are used for diagnosis each time. There are three datasets are set in the experiment, as shown in Table 1. Datasets A, B and C are data sets under the load of 1 hp, 2 hp and 3 hp respectively. Each data set contains 6600 training samples and 1000 testing samples.

**Table 1:** Description of experimental dataset.

Fault type		Normal	IF	IF	IF	OF	OF	OF	RF	RF	RF	Load
Fault number		C0	C1	C2	C3	C4	C5	C6	C7	C8	C9	
Damage diameter(inch)		0	0.007	0.014	0.021	0.007	0.014	0.021	0.007	0.014	0.021	
A	train	660	660	660	660	660	660	660	660	660	660	1
	test	100	100	100	100	100	100	100	100	100	100	
B	train	660	660	660	660	660	660	660	660	660	660	2
	test	100	100	100	100	100	100	100	100	100	100	
C	train	660	660	660	660	660	660	660	660	660	660	3
	test	100	100	100	100	100	100	100	100	100	100	

### 4.2 Experiments settings

In this paper, three groups of experiments are set up to verify the effectiveness and robustness of the proposed ACGAN-SDAE model from unbalanced fault sample size, different signal-to-noise ratio and across different load domains. Therefore, we design three kinds of experiment data settings:

#### 4.2.1 Unbalanced fault sample size dataset

Only part of the data is used for training. Supervised learning needs a huge number of training data to achieve good performance. However, in fact, we usually cannot get enough fault samples to train a deep learning model. Therefore, it is necessary to study the robustness of different models under small fault data. In our experiments, the unbalance rate of training samples in each state mode of data set A is 100%, 40%, 20%, 10% and 5% for comparison experiments.

#### 4.2.2 Different signal-to-noise ratio dataset

In actual fault diagnosis, the sample signal usually contains a lot of noise, which makes the diagnosis performance of the model unsatisfactory. Therefore, in our experiments, Gaussian noise with different signal-to-noise ratio (SNR) from -6 to 10dB is added to data set A to test the recognition rate of the model, so as to verify the anti-noise performance of the model. SNR is defined as follows:

$$SNR_{dB}=10\log_{10}\left(\frac{P_{signal}}{P_{noise}}\right)$$ (13)

where *P*_*signal*_ and *P*_*noise*_ are the power of signal and nosie respectively.

#### 4.2.3 Across different load domains dataset

**Fig 10:**
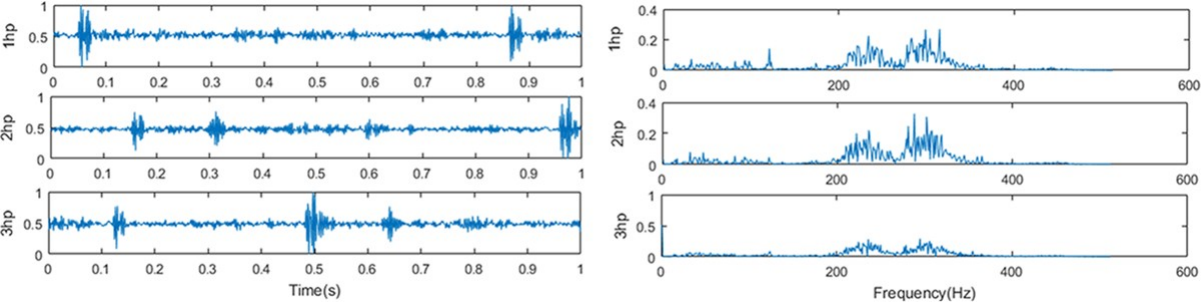
The diagnostic signal with an inner race fault size of 0.014 inches under different loads. (a) the time-domain waveform and (b) the corresponding frequency spectra.

Across different load domains problem is also called cross load domain adaptive problem. **Fig 10** shows the time-domain waveform and frequency spectrum of the diagnostic signal with an inner race fault size of 0.014 inches under different loads. It can be seen from Fig 10 that under different loads, the time-domain and frequency spectrum features of the vibration signal are very different, which will cause the classifier to fail to correctly classify the extracted features, thereby reducing the fault recognition rate. Therefore, it is of great practical significance to use the diagnostic model trained with the data under a load to diagnose the vibration signal when the load changes. The ACGAN-SDAE model will be trained using samples with loads of 1hp, 2hp and 3hp respectively, and the signals under the other two loads will be used as the test set for testing. Detailed description of the across different load domains data is shown in Table 2.

**Table 2:** Description of across different load domains data.

Dataset types	Training set	Testing set	
Description	labeled signals under one signal load	unlabeled signals under another load	
Dataset	Training set A	Testing set B	Testing set C
	Training set B	Testing set C	Testing set A
	Training set C	Testing set A	Testing set B

All the network models used in this paper are trained under the Ubuntu 16.04 operating system and Keras deep learning framework. The CPU model is Intel(R) Core(TM) i7-8700, 16GB memory, the graphics card model is NVIDIA GeForce GTX 1080 Ti. The algorithm is implemented through Python3.6 programming language.

### 4.3 Experimental design and result analysis

#### 4.3.1 Noise factor selection of ACGAN-SDAE

The diagnostic performance of ACGAN-SADE is mainly affected by the discriminator SDAE, and the selection of noise factor *ρ* directly affects the performance of SDAE. The noise factor of SDAE is expressed as the random zeroing ratio of input data. The noise factor is too large or too small will make the SDAE performance decline. If the noise factor is too large, the input signal will be masked by the noise, so that the SDAE can not extract rich fault features. If the noise factor is too small, the sample damage is too little, which leads to poor anti-noise performance. In this section, we have optimized the noise factor *ρ*.

**Fig 11:**
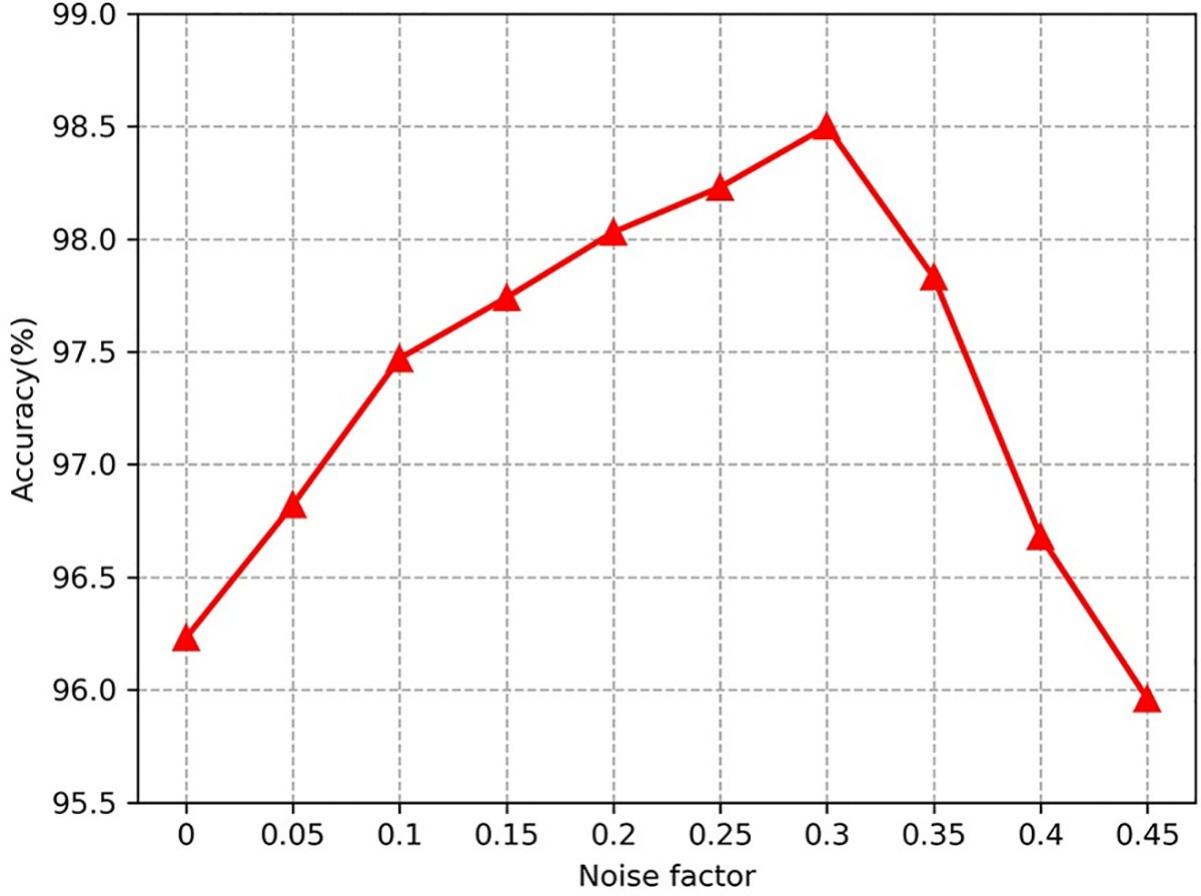
Diagnosis accuracy of ACGAN-SDAE under different noise factors.

Here, ten noise factors of different sizes are studied to determine the best noise factor. To eliminate the effect of contingency, ten tests were carried out, and the average values were taken as the results, the specific results are shown in **Fig 11**. It can be seen that with the increase of noise factor, the diagnostic accuracy presents an "arch" distribution, which is consistent with the previous analysis. The highest diagnostic accuracy is 98.5% when the noise factor is 0.3. Therefore, the noise factor is finally selected as *ρ* = 0.3.

#### 4.3.2 Comparison of diagnostic performance under different fault sample sizes

In this section, unbalanced fault sample size data set will be used for comparison experiments, and the *SNR* = −4*dB* Gaussian noise will be added to data set to simulate the operating environment under noise interference. We compared the diagnostic accuracy of our method with GAN-SAE, SAE, SDAE, MLP and SVM respectively. Among them, the generator of GAN-SAE uses BPNN with a 256-512-1024 structure, and the discriminator uses SAE, which structure is 1024-512-256-10. Both SAE and SDAE are 4-layer with the structure of 1024-512-256-10. The noise factor of SDAE is 0.3. The inputs of GAN-SAE, SAE and SDAE are all spectrum samples. The kernel function of SVM adopts Radial Basis Function (RBF), and the penalty factor and related parameters of the kernel function are optimized through the method of cross validation.The number of hidden nodes set by MLP is 50. And 59 artificial extracted features [31] are selected from time domain and frequency domain as inputs to shallow model SVM and MLP.

**Fig 12:**
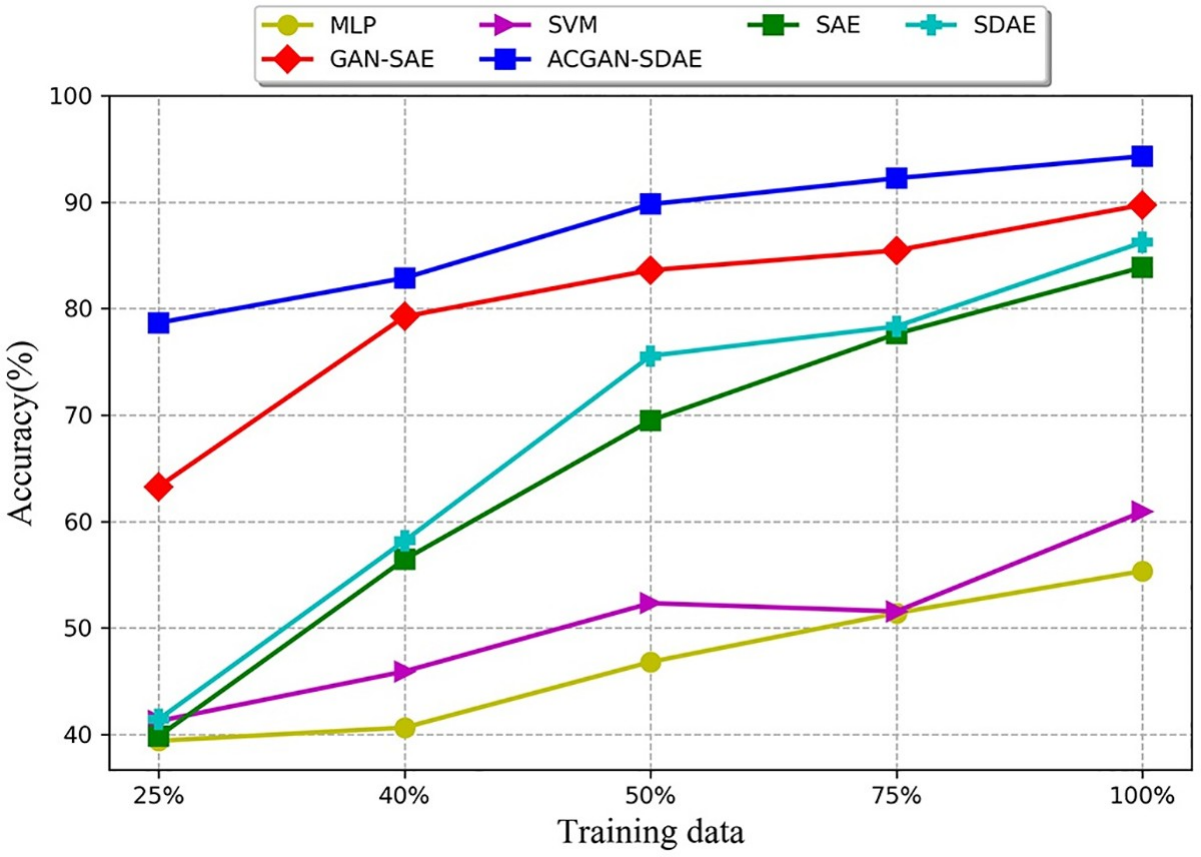
Diagnosis results of different models in different fault sample proportions.

In order to decrease the influence of randomness, repeat the experiment ten times and use the average value as the final diagnosis result. The diagnosis results of different models under different fault sample sizes are given in **Fig 12** and Table 3. As can be seen from **Fig 12** and Table 3, with the increase of the proportion of fault samples, the diagnosis accuracy of deep network model is significantly improved, while the diagnosis accuracy of shallow model has little change. This is because the deep network model can mine rich information from big data, which significantly improve the diagnostic accuracy of the model. In addition, the diagnostic accuracy of GAN-SAE is higher than the SAE and SDAE, which indicate that GAN can improve the generalization ability of the model under limited fault sample size. Under different fault sample sizes, ACGAN-SDAE obtains better results than other comparison methods. In addition, although only 25% of the fault samples were used in our method, the accuracy can reach 78.67%, which proved the good robustness of our method. ACGAN-SDAE combines the generator and discriminator with the adversarial learning mechanism, and use category labels as auxiliary information to enhance the original GAN, improve the generation effect of the generator, and generate high-quality labeled artificial samples to expand the number of fault samples, which greatly improves the fault diagnosis performance in the case of small samples.

**Table 3:** Diagnosis accuracy of different models in different fault sample proportions (%).

Method	25%	40%	50%	75%	100%
MLP	39.40	40.64	46.81	51.40	55.35
SVM	41.25	45.92	52.33	51.56	60.92
SAE	39.82	56.44	69.49	77.63	83.89
SDAE	41.41	58.18	75.56	78.33	86.23
GAN-SAE	63.28	79.26	83.61	85.47	89.75
ACGAN-SDAE	**78.67**	**82.88**	**89.81**	**92.25**	**94.32**

#### 4.3.3 Comparison of diagnostic performance under different SNRs and across different load domains

**Fig 13:**
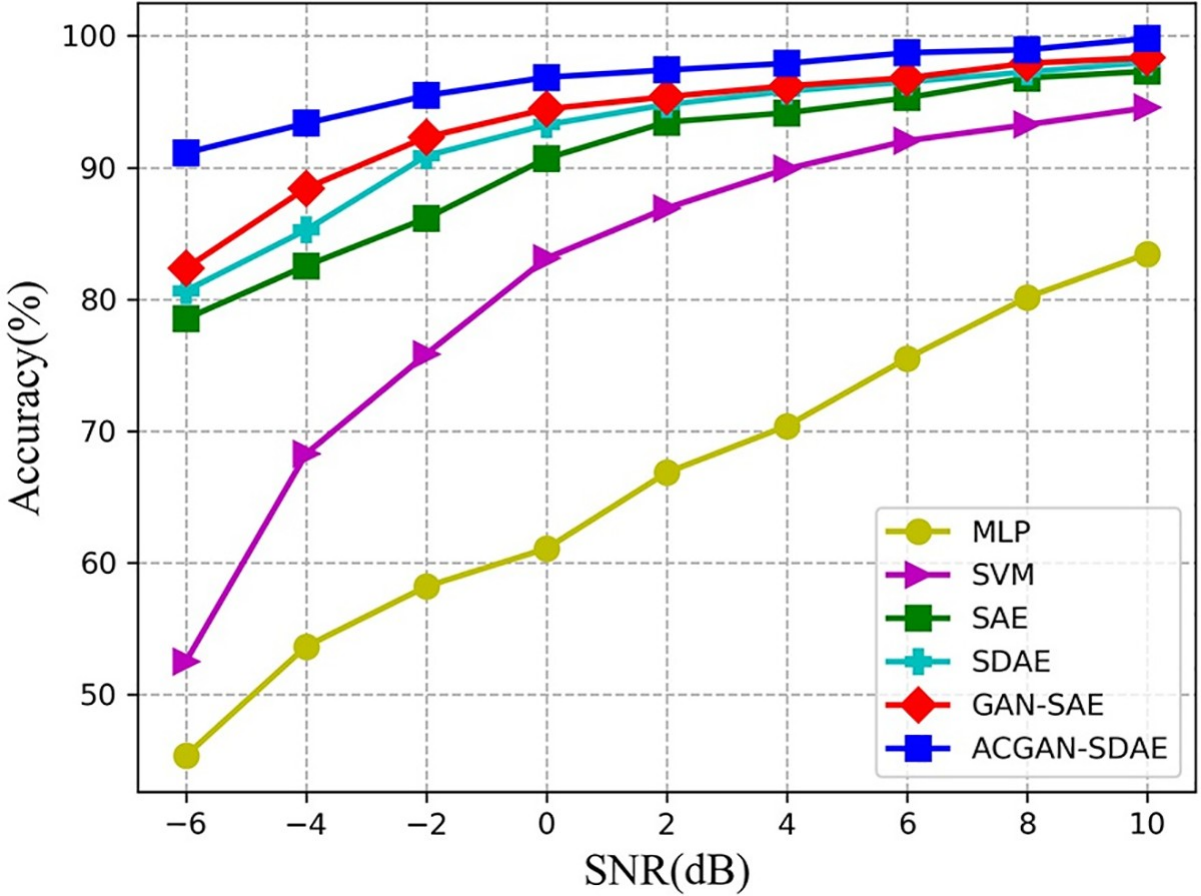
Diagnosis results of six diagnosis models under different SNRs.

In this section, different SNRs data set are used to verify ACGAN-SDAE anti-noise performance. The results are shown in **Fig 13** and Table 4. As can be seen from **Fig 13** and Table 4, with the decrease of SNRs, the diagnostic performance of different models will also decline. This is because with the increase of noise intensity, the sample damage is more serious, which makes it difficult for the model to extract effective features. In addition, although the diagnostic results of SVM are better than MLP, the anti-noise performance of both models is very weak. For example, in the environment with weak noise *SNR* = 4*dB*, the diagnostic accuracy of both models is less than 90%. In contrast, the diagnostic accuracy of ACGAN-SDAE under different SNRs is higher than 90%, and even in the environment with *SNR* = −6*dB*, it is 8.76% and 10.48% higher than GAN-SAE and SDAE, respectively. Benefiting from the adversarial learning mechanism and the denoising principle, ACGAN-SDAE have the best anti-noise robustness in a strong noise environment.

**Table 4:** Diagnosis accuracy of six diagnosis models under different SNRs (%).

Method	SNR(dB)								
	-6	-4	-2	0	2	4	6	8	10
MLP	45.32	53.62	58.17	61.06	66.82	70.37	75.51	80.14	83.45
SVM	52.48	68.25	75.83	83.11	86.92	89.90	92.04	93.26	94.55
SAE	78.51	82.54	86.15	90.70	93.46	94.16	95.30	96.81	97.31
SDAE	80.64	85.26	90.89	93.25	94.79	95.81	96.52	97.26	98.01
GAN-SAE	82.36	88.43	92.31	94.45	95.38	96.20	96.81	97.92	98.35
ACGAN-SDAE	**91.12**	**93.36**	**95.47**	**96.86**	**97.41**	**97.92**	**98.27**	**98.96**	**99.80**

**Fig 14:**
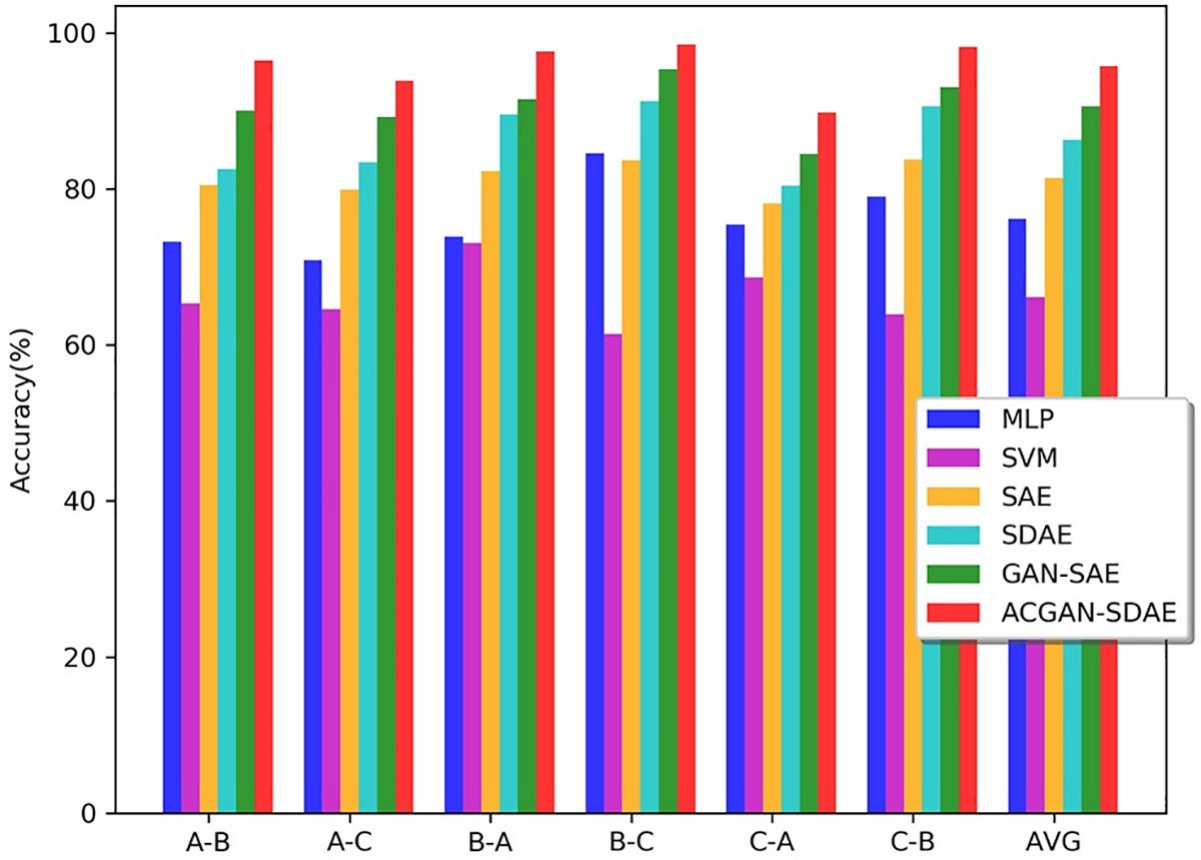
Diagnosis accuracy of different models under across different load domains.

Next, we used across different load domains data set to simulate fault diagnosis under variable working conditions, and further test domain adaptation performance of ACGAN-SDAE. The results are shown in **Fig 14** and Table 5. As can be seen from **Fig 14** and Table 5, the average accuracy of SVM and MLP is less than 80%, the performance of GAN-SAE is better than SAE and SDAE, and the average diagnostic accuracy can reach 90.61%. GAN-SAE can learn more sample features through adversarial training. ACGAN-SDAE helps to understand the original data distribution during the generator’s simulation of the generation of fake data. The adversarial learning can be used as a cross domain regularizer, and the universal and domain-invariant features of data can be better learned. As a result, the model has significant cross domain adaptive ability, with the average accuracy reaching the highest 95.75%. In addition, we can observe that the diagnostic accuracy of all models from C to A and A to C is significantly lower than that of other cross domain conditions. This result is consistent with intuition. When there are big differences between the two working conditions, the diagnostic performance of the model is poor, that is, domain adaptability of the model are not good.

**Table 5:** Diagnosis accuracy of different models under across different load domains (%).

Method	A-B	A-C	B-A	B-C	C-A	C-B	AVG
MLP	73.24	70.89	73.86	84.61	75.47	79.05	76.18
SVM	65.31	64.55	73.05	61.44	68.65	63.92	66.15
SAE	80.53	79.92	82.33	83.65	78.11	83.73	81.37
SDAE	82.51	83.47	89.58	91.28	80.40	90.62	86.31
GAN-SAE	90.02	89.25	91.50	95.31	84.52	93.06	90.61
ACGAN-SDAE	**96.47**	**93.90**	**97.62**	**98.53**	**89.81**	**98.22**	**95.75**

#### 4.3.4 Feature extraction and generate samples visual analysis of ACGAN-SDAE

**Fig 15:**
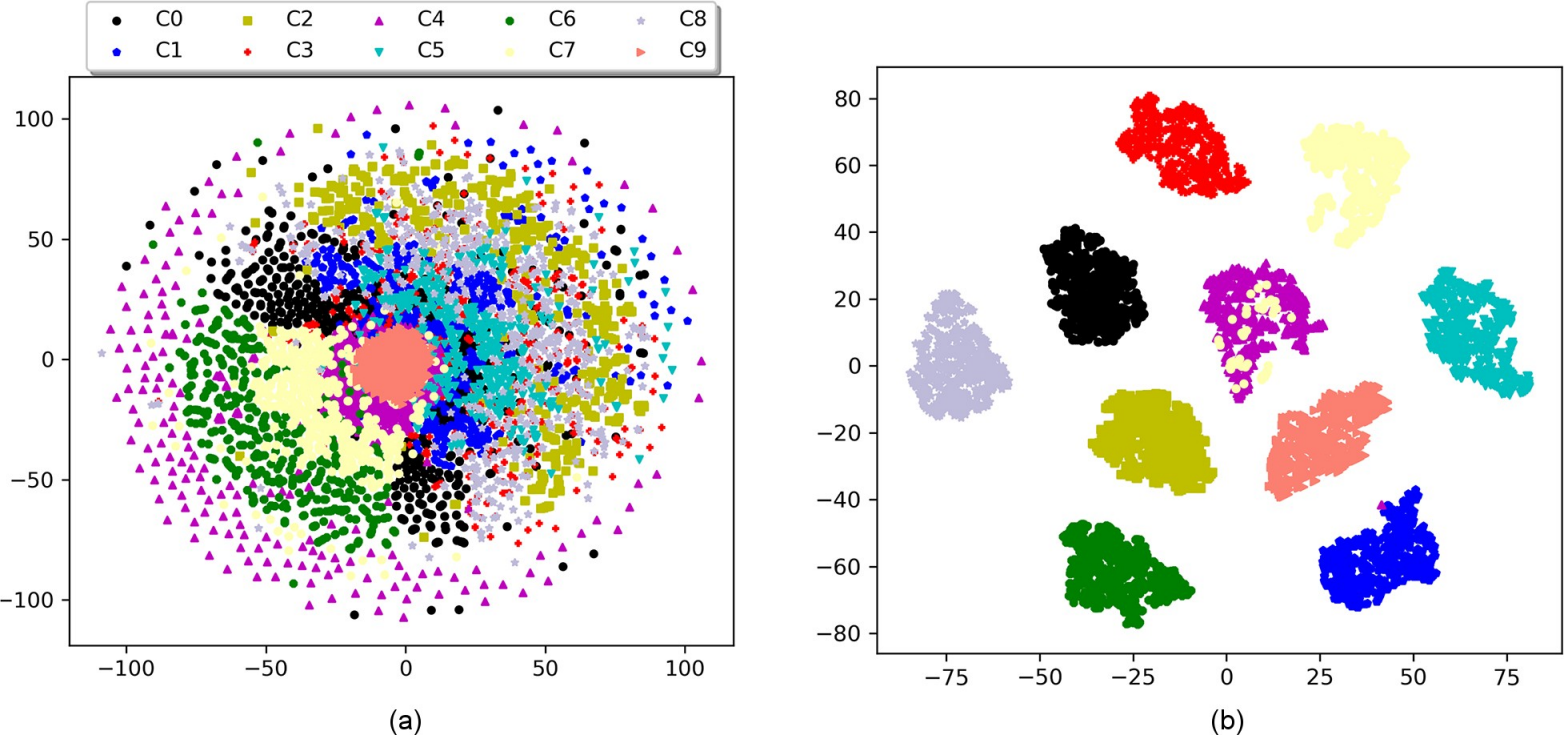
Feature visualization via t-SNE. (a) features of original input data, (b) features extracted by ACGAN-SDAE.

**Fig 16:**
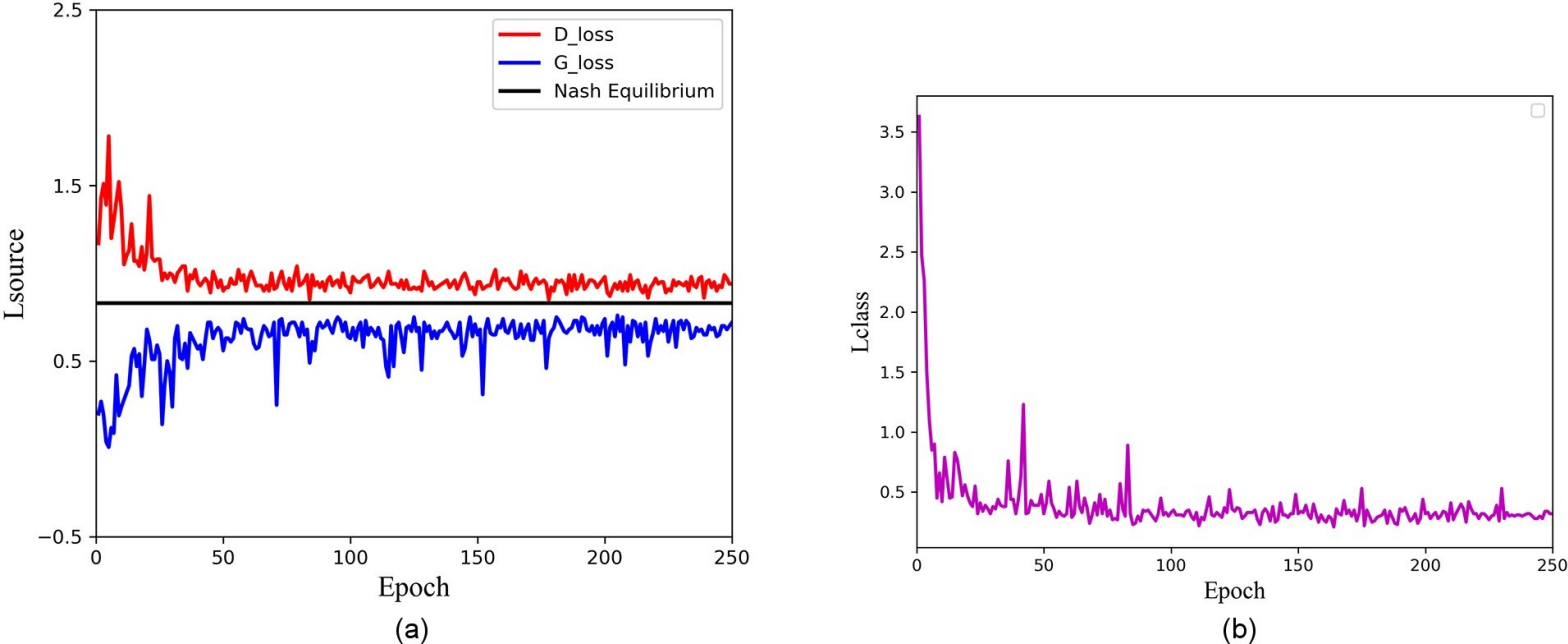
ACGAN-SDAE training loss. (a) adversarial loss, (b) classification loss.

In order to better understand the feature extraction ability of ACGAN-SDAE, by using t-SNE [32] dimension reduction technology, the features of dataset A at the input layer and the last hidden layer of the discriminator SDAE were reduced to two dimensions and visualized. It can be seen from **Fig 15** that the original feature distribution of the input signal is scattered before feature extraction, and different categories are mixed with each other. It is difficult to distinguish them. Through the feature extraction of the discriminator SDAE, we can clearly see that the features of the same fault type have been well aggregated, and the features of different fault types have been well separated. This indicates that ACGAN-SDAE has excellent feature extraction capabilities and can effectively distinguish various fault types. **Fig 16** shows the training loss of confrontation and classification in each epoch. It can be observed that with the increase of the number of epochs, the training loss of the generator and the discriminator gradually converges and finally stays around the Nash equilibrium, and the classification loss also tends to be convergent and stable.

**Fig 17:**
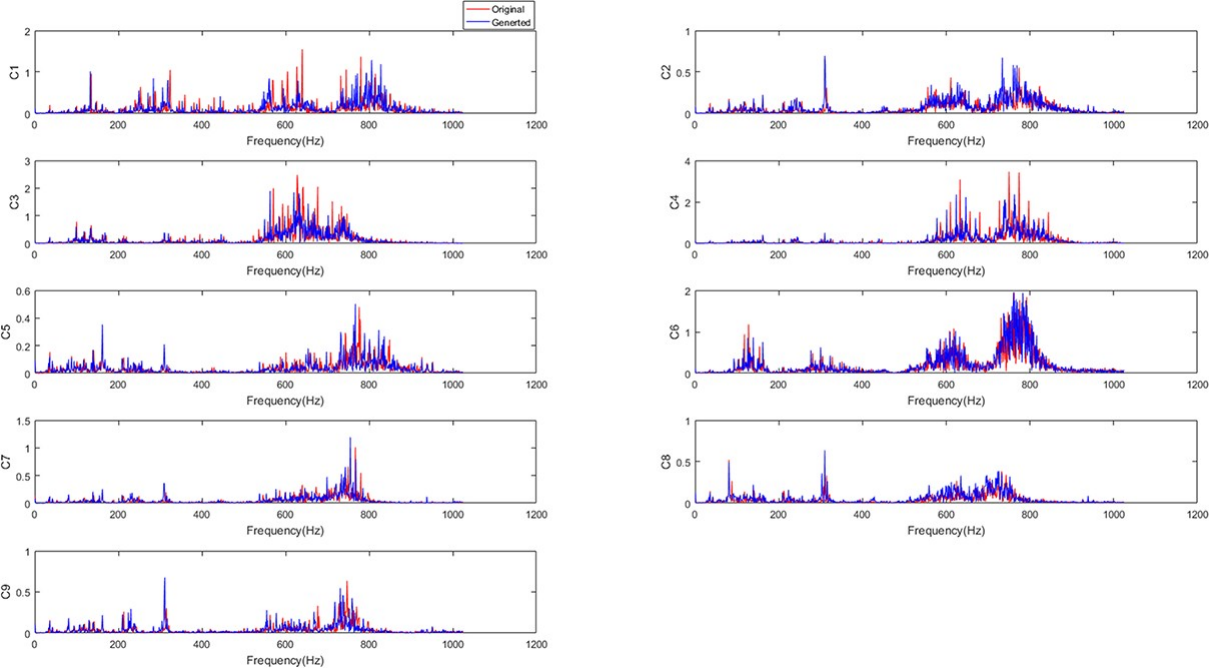
The spectrum of original samples and corresponding generated samples under nine fault conditions.

After training, generated samples from ACGAN-SDAE are obtained. **Fig 17** shows the frequency spectrum of the original samples and the corresponding generated samples under nine fault conditions. From the figure shown, we can see that the original samples and the corresponding generated samples are highly similar, that is, the samples are different but the distributions are similar. Therefore, ACGAN-SDAE can effectively learn the data distribution by adding auxiliary category label information to generate high-quality generated samples similar to the original samples, so as to expand the number of fault samples, and further improve the robustness of the model.

## 5. Conclusions and future work

In this paper, a novel ACGAN-SDAE fault diagnosis method is proposed to solve the problem of data unbalance caused by small sample size, cross domain adaptive problem under variable load and the influence of high background noise pollution in the fault diagnosis of rolling bearing. Through the analysis of the experimental results, the following conclusions can be drawn:

1. ACGAN can adaptively learn the data distribution to generate high-quality artificial samples by adding auxiliary category label information, so as to expand the number of training fault samples and improve the fault feature extraction ability of the model under the condition of small fault samples.
2. SDAE can be used as discriminator to automatically extract features with better robustness, which makes the model have stronger anti-noise capability. At the same time, in the process of simulating the generation of fake data, the generator is helpful to understand the distribution of original data, and the adversarial learning is used as cross domain regularizer to learn the universal and domain-invariant features of data, which makes the model have significant cross domain adaptive ability.
3. Compared with other fault diagnosis models (GAN-SAE, SDAE, SAE, MLP and SVM), ACGAN-SDAE has better diagnosis performance and stronger robustness.

At present, spiking neural P systems(in short, SNP systems) [33] is in full swing, and it is commonly used in the power system fault diagnosis field [34]. SNP systems are a type of membrane computing model, which is abstracted from the neurophysiological behavior of biological neurons sending out electronic pulses along synapses. SNP systems are a distributed and parallel computing model in which neurons work in parallel. Therefore, in the future work, we will apply SNP systems and variant structure to mechanical fault diagnosis to solve the uncertainty and incomplete problems in mechanical fault.

## Supporting information

S1 Data (RAR)
